# Development of a multienzyme isothermal and lateral flow dipstick combination assay for the rapid detection of goose astrovirus II

**DOI:** 10.3389/fcimb.2024.1424212

**Published:** 2024-08-06

**Authors:** Yinchu Zhu, Liu Chen, Xin Xu, Weicheng Ye, Zheng Ni, Suxin Huo, Jionggang Hua, Tao Yun, Huochun Yao, Hongyu Wang, Cun Zhang

**Affiliations:** ^1^ State Key Laboratory for Managing Biotic and Chemical Threats to the Quality and Safety of Agro-products, Institute of Animal Husbandry and Veterinary Sciences, Zhejiang Academy of Agricultural Sciences, Hangzhou, China; ^2^ College of Veterinary Medicine, Nanjing Agricultural University, Nanjing, China

**Keywords:** goose astrovirus, multienzyme isothermal rapid amplification, lateral flow strip, rapid detection, clinic diagnostic

## Abstract

**Introduction:**

Goose astrovirus (GAstV) is a newly emerging pathogen that is currently widespread among geese, causing visceral gout and leading to substantial gosling mortalities, posing a severe threat to the waterfowl industry. GAstV II is the predominant epidemic strain, characterized by its high morbidity and mortality rate. Consequently, there is an urgent necessity to develop an effective diagnostic approach to control the dissemination of GAstV II, particularly in clinical farms with limited laboratory resources.

**Methods:**

In this study, a novel multi-enzyme isothermal rapid amplification (MIRA) and lateral flow dipstick (LFD) combined assay was developed. Different primers designed specific targeting a highly conserved region within the viral RdRp gene for the detection of GAstV II. Primers optimized and MIRA-LFD assay analyzed its performance regarding limits of detection, specificity, and efficiency of detection.

**Results:**

The developed MIRA amplification is conducted at a constant temperature and accomplished within 10 minutes. Subsequent naked-eye observation of the LFD strips merely takes 5 minutes. The established MIRA-LFD method exhibits high specificity, with no cross-reaction with other pathogens and attains a detection sensitivity of 1 copy/μl, which is consistent with the reverse transcription quantitative PCR (RT-qPCR) assay. Further evaluation with clinical samples indicates that the accuracy of this MIRA-LFD method correlates well with RT-qPCR for the detection of GAstV II.

**Conclusion:**

In summary, the convenience, sensitivity, and rapidity of this newly developed detection method offer a significant advantage for on-site diagnosis of GAstV II.

## 1 Introduction

The Astrovirus are recognized as significant viral pathogens that primarily responsible for enteritis diseases in humans and various other mammals, nephritis in chickens and pigeons, hepatitis in ducklings, and encephalitis in humans, cattle, and sheep (Snodgrass and Gray, 1977; Koci and Schultz-Cherry, 2002; Mittelholzer et al., 2003; Zhang et al., 2017). The astroviridae family is generally divided into two genera, Mamastrovirus and Avastrovirus, based on whether the astroviruses (AstVs) are isolated from mammals or avians, respectively (Madeley and Cosgrove, 1975). Since 2015, novel goose astroviruses (GAstVs) have emerged and rapidly spread across China including Heilongjiang, Shandong, Henan, Hunan, Anhui, Jiangsu, Zhejiang, Jiangxi, Fujian and Guangdong provinces (Zhang et al., 2017; Chen et al., 2020a; Li et al., 2024). This widespread distribution poses a significant threat to the healthy development of China’s poultry farming industry. Compounding the challenge is the absence of a commercial vaccine or targeted drug against this disease, making prevention and control efforts difficult.

In contrast to previously reported avian AstVs, GAstV primarily manifests as gout and fatal outcomes in goslings, characterized by severe urate deposition in the viscera and other interstitial tissues, particularly in the heart, liver, and kidney (Zhu et al., 2022). Thus, GAstV leads to mortality rates of approximately 50% in goslings aged 5 to 20 days. Notably, several reports have revealed GAstV in adult goose, Cherry Valley ducklings and Muscovy ducklings, indicating a diverse host range and suggesting a potential risk of intra- and cross-species transmission (Zhang et al., 2021; Fei et al., 2022; Fu et al., 2022; Wang et al., 2022a). Although the GAstV comprises two genotypes GAstV I and GAstV II, clinical epidemiological investigations have consistently shown that GAstV II strains predominate in most samples. These strains have been identified as the primary cause of urate deposition in viscera and hemorrhage in goslings. Additionally, GAstV I strains are often found in co-infection with GAstV II strains (Wang et al., 2022a). Consequently, the development of a sensitive and specific detection method for GAstV II is crucial for clinical investigations and disease control efforts.

GAstVs are small non-enveloped viruses with positive-sense, single-stranded RNA (+ssRNA) genomes, which are approximately 7kb in size (Cortez et al., 2019). The genome structure contains a 5’ and 3’ untranslated region (UTR), three open reading frames (ORFs; ORF1a, ORF1b, and ORF2), and a poly(A) tail (Zhu and Sun, 2022). ORF1a encodes a non-structural protein spanning 1085 amino acids, which includes transmembrane (TM) helical motifs and a 3C-like serine protease. ORF1b encodes an RNA-dependent RNA polymerase (RdRp), a region highly conserved within the same species. ORF2 encoded the structural capsid protein essential for virion assembly, contain a conserved N-terminal capsid core and a highly variable C-terminal spike domain. In this study, the RdRp gene will be selected as the detection target, owing to its stability and conserved nature within the GAstV II genome.

Several diagnostic methods have been developed for detecting and identifying GAstV infections, including real-time polymerase chain reaction (RT-PCR), quantitative RT-PCR (RT-qPCR), which are widely utilized molecular detection techniques in clinical settings (Wan et al., 2019; Yang et al., 2020; Yin et al., 2020). Additionally, immune-related detection methods such as indirect and competitive enzyme-linked immunosorbent assay (ELISA) were have been established (Wang et al., 2022c; He et al., 2023; Zhang et al., 2023). While these assays play a crucial role in GAstV detection, their widespread application is hindered by the requirement for trained technicians, complex and costly instruments, and time-consuming procedures. Consequently, these methods are not suitable for use on farms or low-resource laboratories. Therefore, there is a pressing need for a rapid, reliable, simple, and isothermal amplification-based assay to bridge the technological gap in GAstV detection on farms.

Isothermal nucleic acid amplification technologies have undergone rapid development in recent years, offering the advantage of operating at a constant temperature within a short timeframe of around 20 minutes (Sun et al., 2021; Wang et al., 2023). This eliminates the need for a thermal cycler, making these methods convenient and rapid tools for pathogen detection. Various isothermal amplification-based techniques have been established to date, including loop-mediated isothermal amplification (LAMP), recombinase polymerase amplification (RPA), and multi-enzyme isothermal rapid amplification (MIRA), all capable of operating at a constant temperature (Li et al., 2022). MIRA, akin to RPA in its principle, represents a novel rapid isothermal amplification technique for nucleic acid detection (Piepenburg et al., 2006). The amplification products can be verified through gel electrophoresis, visualized using lateral flow dipstick (LFD), or detected using fluorescence signal analysis (Heng et al., 2022; Liu et al., 2022). MIRA-LFD, a variant of MIRA detection, enables rapid visual readout. In this method, the colloidal gold strip relies on the activity of the nfo enzyme, and the final result can be detected using colloidal gold technology employing the sandwich hybridization method, which involves the addition of specifically designed molecular probes according to the template.

In this study, a MIRA-LFD combination assay targeting the conserved region of GAstV II for rapid and reliable virus detection was established. Our aim was to develop a new method that maintains consistency with real-time PCR while significantly reducing the reaction time. This approach provides obvious advantages for detection in the fields settings or laboratories with limited resources, offering an alternative tool for disease diagnosis and prevention.

## 2 Materials and methods

### 2.1 Viruses strains and plasmids

The novel goose astrovirus strains including GAstV I (ZJC14) and II (ZJLD) were isolated from the kidneys of diseased geese exhibiting symptoms of gout (Wang et al., 2022a). Additionally, various other avian viruses, such as goose parvovirus (GPV) strain, tembusu virus (TMUV) strain, Newcastle disease virus (NDV), avian influenza virus (AIV), duck enteritis virus (DEV), classical duck reovirus (CDRV) and novel duck reovirus (NDRV)strains, were maintained in our laboratory and stored at −80°C until use. The viral RNA was extracted from the cell cultural using a Takara Virus DNA/RNA Kit (Takara, Dalian, China) in accordance with the manufacturer’s instructions. Subsequently, the ORF1b gene of GAstV II was amplified with RT-PCR and cloned into the pEASY-Blunt plasmid with Transgene kit (Beijing, China), resulting in the construction of pEASY-Blunt-RdRp. The concentration of recombinant plasmids were determined by an ND-2000c spectrophotometer (NanoDrop, Wilmington, USA). To quantify the copy number of pEASY-Blunt-RdRp, the formula was employed: number of plasmids in copies/µL = (6.02×10^23^) × (plasmid concentration in ng/µL)/(genome length in bp) × (10^9^) × 660. Serial 10-fold dilutions ranging from 1 × 10^8^ to 0.1 copies/µL were prepared and stored at -80°C for use in subsequent assays.

### 2.2 Design and synthesis of MIRA primers and probe

**Figure 1:**
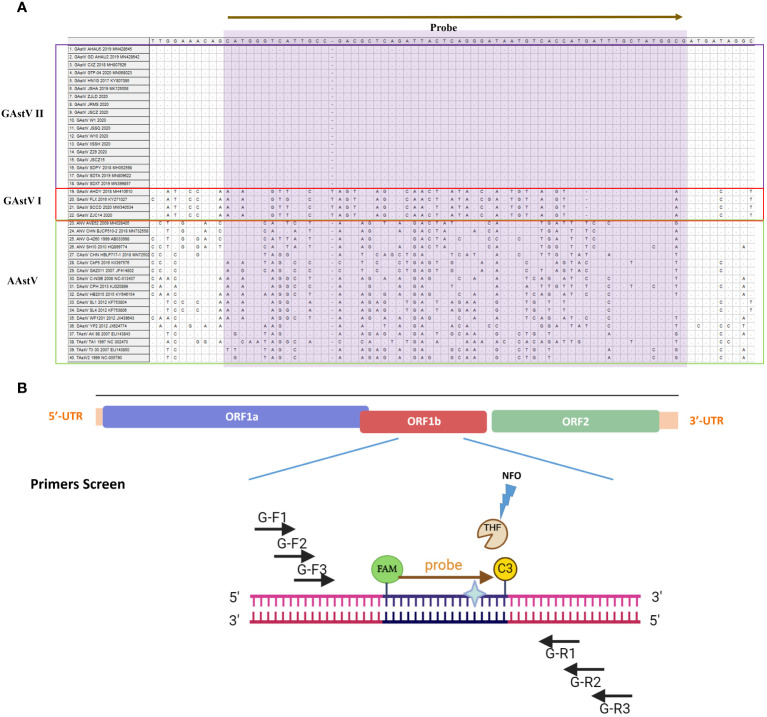
Schematic diagram of the primer design for MIRA. **(A)** The information different avian astrovirus were list, the name of isolates of the sequences are indicated on the left. Nucleotide residues that match the majority are indicated by dots. The conserved sequence of GAstV II for primers design were selected. **(B)** The primes targeting to the conserved ORF11b region of GAstV II. Based on the principles described above, the MIRA primers with the target size of 100-200 bp were shown. Three forward and three reverse candidate primers that surround the probe are indicated with arrows of different lengths. Forward primers, G-F1,G-F2, G-F3, Forward primers, Reverse primers, G-R1, G-R2, G-R3. The probe with N-FAM and C-C3Space.

The full length ORF1b gene sequences of GAstV II from diversity isolates were downloaded in GenBank and aligned, the information of AAstV are list in **Supplementary Table S1**. The N-terminal region of the gene showed high conservation, making it an ideal target sequence for primer and probe design. Following the guidelines provided by AMP-Future Biotech company (Changzhou, China), we designed MIRA primers targeting the ORF1b gene. To combine LFD detection with the MIRA assay, we constructed a sequence complementary to the target fragment between the upstream and downstream primers as a probe (**Figure 1**). The probe length ranged from 25–35bp nucleotides, while the expected amplicon size was between 150-300bp. An antigen marker (FAM) was added to the 5’-end, with tetrahydrofuran (THF) labeled in the center of the sequence, and a C3-spacer was added to the 3’-end. Three upper primers and three down primers were designed to match probe. The sequences of the MIRA primers and probe for GAstV II detection were listed in **Table 1**. These sequences were synthesized by Sangon Biotech Company (Shanghai, China) and purified with high-performance liquid chromatography (HPLC).

**Table 1:** The primers and probes used in this study.

Prime	Sequence(5’-3’)
G-F1:	CCAACTGGGGAGGTTTGTACAGTTAAGAAGG
G-F2:	GAAGGGAAATCCAAGTGGCCAATATTCAAC
G-F3:	CAATATTCAACAACAGTGGACAACAATATGTG
G-R1:	Biotin-TATACATATCTATGACGACATTCGTGTCGT
G-R2:	Biotin-CCTTGACATTTTCTGGTTTAACCCACATACC
G-R3:	Biotin-GAAAGTCATACCCTCAATATCATCAGAGA
GAstV-Probe:	FAM-CATGGGTCATTGCCGACGCTCAGATTACTCA[THF]GGATAATGTCACCATG-C3Spacer
GAstV II-probe	FAM-ATGAAGCAACAGACAGAACGGCGG-BHQ1
GAstV II-F	GAGCAGGACCAGAATGAGAAA
GAstV II-R	CACCACCAATGAGCCTAGATAC

### 2.3 MIRA reaction

The MIRA was performed using the RNA isothermal rapid amplification kit (#WLB8201KIT, AMP-Future Biotech Co. Ltd), according to the manufacturer’s instructions. To screen for the most suitable primer pairs, different combinations were designed. For the basic MIRA assay, a 50ul reaction mixture contained 29.4 µL A buffer, 2.5µL B buffer, 2 µL of each forward/reverse primer (10 µM), 2 µL RNA template and remaining volume filled with ddH_2_O. Then, the reaction tube was gently shaken upside down 10 to 15 times ensure thorough mixing of the reagents before being incubated at 42°C for 12 minutes. The generated products were purified with phenol followed by centrifugation at 5,000 rpm for 5 minutes and separated on a 2% agarose gel through electrophoresis. After electrophoresis, the products were visualized using an automatic digital gel image analysis system (BioRad, California, USA). This allowed for the detection and analysis of the amplified products.

### 2.4 MIRA-LFD assay

MIRA-LFD was conducted using an amplification kit (Colloidal gold test strip type) (#WLRN8209KIT AMP-Future Biotech Co. Ltd, Changzhou, China), according to the manufacturer’s instructions. In this assay, 0.6 μL of nfo probe (10 mM) was added to the basic MIRA reaction mixture and the reactions were performed at 42°C for 12 minutes. Finally, the generated products were diluted 1:20 with H_2_O. Aliquots of the diluted products (50ul) were applied to LFDs (#WLFS8201 AMP-Future Biotech Co. Ltd, Changzhou, China), and the LFDs were then visually observed within 5 minutes based on the appearance of quality control and detection lines. This allowed for rapid and convenient detection of the amplified products directly on the LFDs.

### 2.5 Optimum temperature and time for the MIRA assay

To determine the optimal MIRA amplification system, a gradient optimization was performed across a range of reaction temperatures and incubation times. The reaction temperatures tested included 20°C, 25°C, 30°C, 37°C, 42°C and 45°C. Additionally, incubation times of 4, 6, 8, 10, and 12 minutes were evaluated to optimize detection. By systematically varying these parameters, we aimed to identify the combination of temperature and incubation time that yielded the highest amplification efficiency and specificity for GAstV II detection.

### 2.6 Specificity analysis of the MIRA assay

To evaluate the specificity of this newly developed MIRA-LFD assay, the nucleic acid extracted from preserved reference viruses of GAstV I, TMUV, CDRV, NDRV, NDV, GPV, AIV and DEV, which as important virus affecting waterfowl, were utilized for analysis. Meanwhile, RNase-free distilled water (ddH_2_O) as the negative control. By comparing the results obtained from the MIRA-LFD assay with the nucleic acids extracted from these reference viruses and the negative control, we can determine the specificity of the assay in accurately detecting GAstV II without cross-reactivity with other common waterfowl viruses.

### 2.7 Sensitivity and repeatability analysis of the MIRA assay

The sensitivity of the MIRA-LFD assay was determined using the standard plasmid PEASY-Blunt-RdRp, which was constructed and preserved in our laboratory. Serial of 10 dilutions in ddH_2_O (diluted from10^8^ to 0.1 copies/uL) were used and RNase-free ddH_2_O as the negative control were prepared for analysis. The sensitivity detection by RT-qPCR were conducted using the Taq Pro HS Probe Master Mix RT-qPCR kit according the manufacturer’s protocol (Vazyme, Nanjing, China). The RT-qPCR amplification assay was also employed to detect the positive standard plasmid established by our lab. The optimal qPCR system included the following components: 10μl of 2×Premix, 0.4μl of forward primer (10mM), 0.4μl of reverse primer (10mM), 0.2 μl of probe (10mM), 0.4μl of ROX Reference Dye II, 2μl of standard plasmid with serial dilutions, and 6.6μl of nuclease-free H_2_O.The reaction conditions were set at 95°C for 30s, followed by 40 cycles of 95°C for 10s, 60°C for 30s. The amplification curve was generated by plotting the logarithm of the plasmid copy number against the measured cycle threshold values. Nuclease-free water was used as the negative control in the RT-qPCR assay. All reactions were performed in triplicates. The qPCR was conducted using the Applied Biosystems QuantStudio5 system (Thermo Fisher Scientific., USA).

### 2.8 Clinical sample performance evaluation

A total of 90 samples including tissue samples (liver and kidney) and nasal swab from southeast China were collected between 2021 and 2023 (**Supplementary Table S2**). The collected tissue samples were homogenized in phosphate-buffered saline (PBS) and centrifuged at 10,000 ×g for 15 minutes. This process resulted in clarified suspensions, which were then used for RNA extraction. All the samples were processed as clarified suspensions for RNA extraction. The extracted RNA from all samples was subjected to amplification using both the MIRA-LFD assay and the RT-qPCR assay. By comparing the results obtained from these two assays, we aimed to evaluate the performance and reliability of the MIRA-LFD assay for the detection of GAstV II in the collected samples.

### 2.9 Ethics statement

All animal samples mentioned in the experiment were conducted in accordance with the Guidelines for Experimental Animals of the Ministry of Science and Technology (Beijing, China) and were approved by the Institutional Animal Care and Use Committee (IACUC) of Zhejiang Academy of Agricultural Sciences (protocol code 2023ZAASLA10).

## 3 Results

### 3.1 Primer screening and identification

In this study, an ideal exo probe was designed within the conservative region of the GAstV II RdRp gene (**Figure 1**). Then three upstream and three downstream candidate primers were designed to flank the probe region. Given the critical role of primers in the MIRA process, a series of primer screening experiments were conducted according to the manufacturer’s instructions.

**Figure 2:**
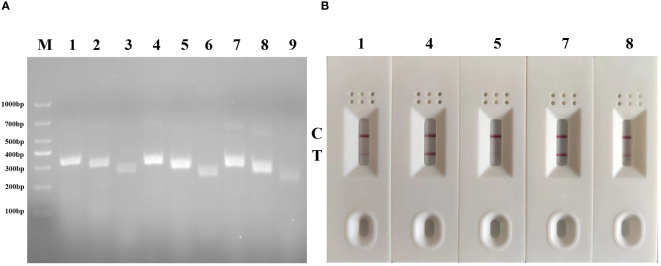
Primer screening tests of MIRA and MIRA-LFD for GAstV II detection. **(A)** Agarose gel electrophoresis of 9 groups MIRA primers amplification products. M, 1,000 DNA marker; 1: G-F1/G-R3,2: G-F1/G-R2, 3: G-F1/G-R1, 4:G-F2/G-R3,5: G-F2/G-R2, 6: G-F2/G-R1, 7: G-F3/G-R1, 8: G-F3/G-R2, 9: G-F3/G-R1. **(B)** Strip change for MIRA-LFD amplified products. C, control line, T, test line.

Totally, 9 pairs primers of forward and reverse primers were screened and the gel imaging results demonstrated successful amplification of fragments with the expected size for all primer pairs. Among these combinations, five pairs primers (G-F1/G-R3, G-F2/G-R3, G-F2/G-R2, G-F3/G-R3, G-F3/G-R2) yielded the most robust amplification with bright bands on the agarose gel, indicating high amplification efficiency compared to other combinations (**Figure 2A**). They would be regarded as the optimal primer pair. Among them, primer G-R3 exhibited the highest efficiency. Subsequently, the primers that demonstrated the best amplification performance were subjected to a second screening using MIRA-LFD. The results confirmed that the primer pair G-F2/G-R3, along with its corresponding probe, exhibited faster amplification and produced a clear test line (**Figure 2B**). Therefore, the combination of primer pair G-F2/G-R3 with its probe was selected as the optimal primer set and used in the subsequent MIRA-LFD assays.

### 3.2 Optimization of the reaction temperature and time

**Figure 3:**
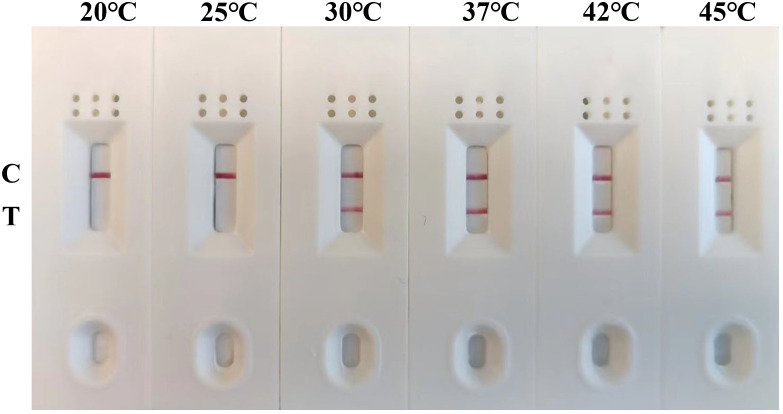
Optimization of the reaction temperature for the MIRA-LFD assay. The optimal amplification reaction time was determined by examining various temperature settings ranging from 20°C, 25°C, 30°C, 37°C, 42°C and 45°C. C, control line; T, test line.

**Figure 4:**
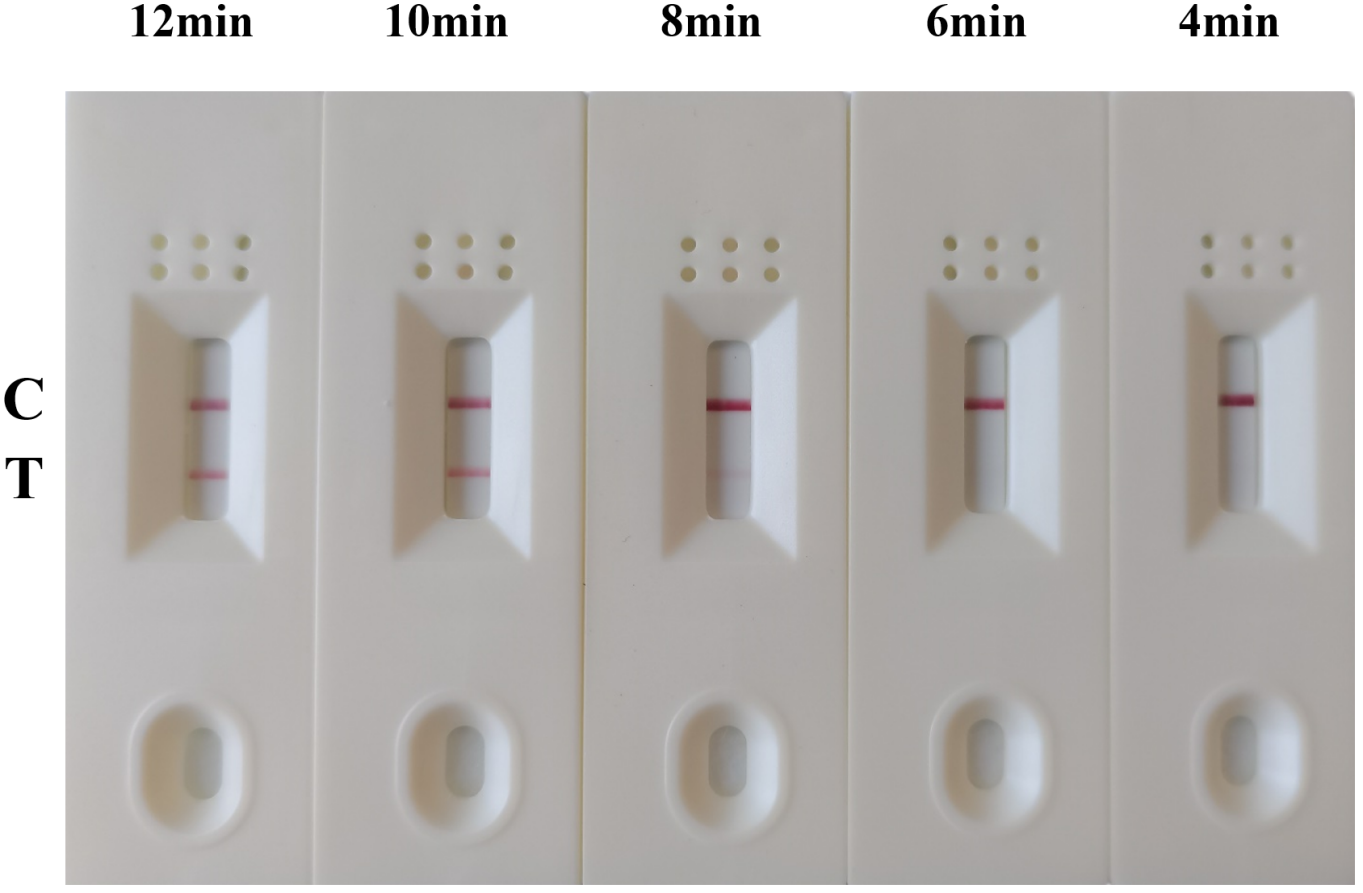
Optimization of the reaction time for the MIRA-LFD assay. The optimal amplification time was determined by examining different time: 4 min, 6 min, 8 min, 10 min and 12 min. C, control line; T, test line.

To determine the optimal amplification temperature and the shortest amplification time, the MIRA-LFD assay was performed according to its manufacturers. And we performed the assay using six temperature gradients (20, 25, 30, 37, 42, and 45°C) for a 12-minute reaction period. The results indicated that the best performance was observed between 30°C and 45°C, with visible test lines on the lateral flow dipsticks across this temperature range (**Figure 3**). Considering these results and the manufacturer’s instructions, we selected 42°C as the optimal reaction temperature for subsequent experiments. Next, various reaction times (4, 6, 8, 10 and 12 minutes) were evaluated to determine the shortest amplification time required for the MIRA-LFD response. The results showed that a visible test band could be observed on the colloidal gold strip after 10 minutes of amplification (**Figure 4**). Furthermore, the test line was most pronounced after 10 minutes of reaction, comparable to the 12 minutes reaction. Consequently, the entire testing process, including MIRA amplification and lateral flow dipstick detection, could be completed in just 15 minutes or less, providing rapid and efficient detection of GAstV II RdRp.

### 3.3 Specificity determination of MIRA-LFD assay

**Figure 5:**
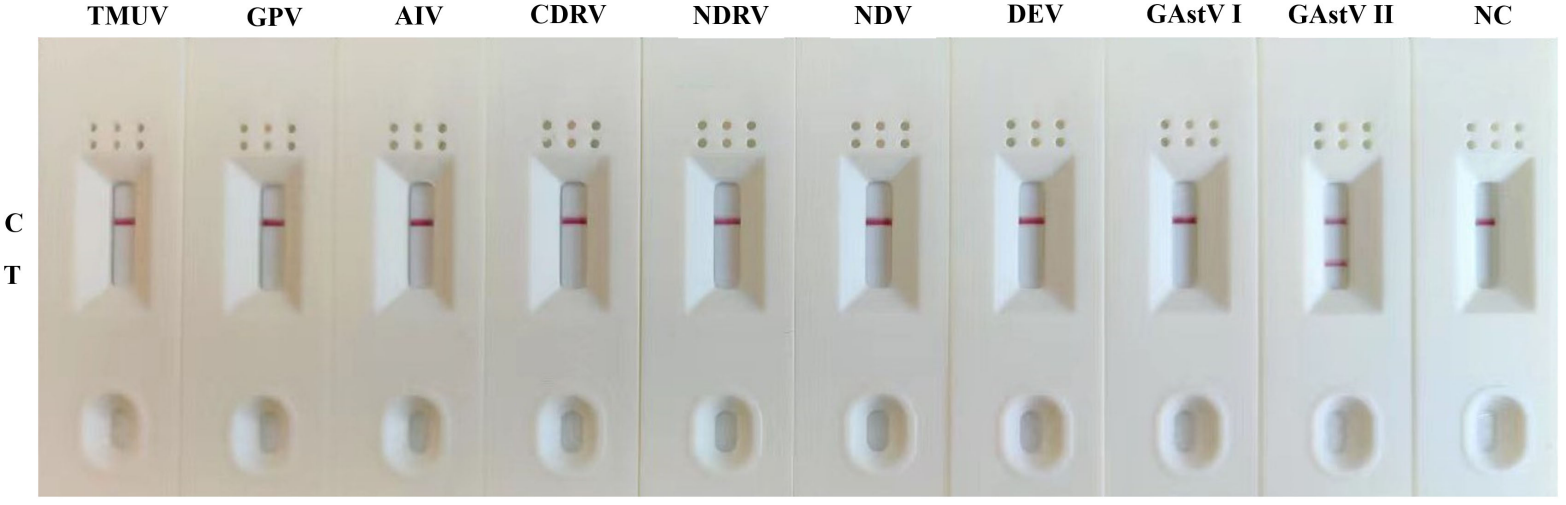
Specificity of the MIRA-LFD assay. The only the positive sample GAstV II produced amplification signals, whereas other waterfowls viruses produced no amplification signals. NC, negative control; C, control line; T, test line.

The specificity analysis of the GAstV II MIRA-LFD assay was performed using the nucleic acids extracted from various viruses, including GAstV II, GAstV I, TMUV, GPV, AIV, CDRV, NDRV. NDV, and DEV as templates. The results of the specificity test revealed that only when the GAstV II nucleic acid was used as template, both the control and test lines were observed in the MIRA-LFD detection (**Figure 5**). No test lines were produced when other virus samples or negative controls were used. Furthermore, the control line on the colloidal gold test strip was clearly visible, indicating the integrity of the experiment. Thus, these results demonstrating that established MIRA-LFD assay for GAstV II exhibits reasonable specificity and does not cross-react with other waterfowl viral samples. This specificity is crucial for accurate and reliable detection of GAstV II in clinical samples.

### 3.4 Sensitivity determination of MIRA-LFD assay

**Figure 6:**
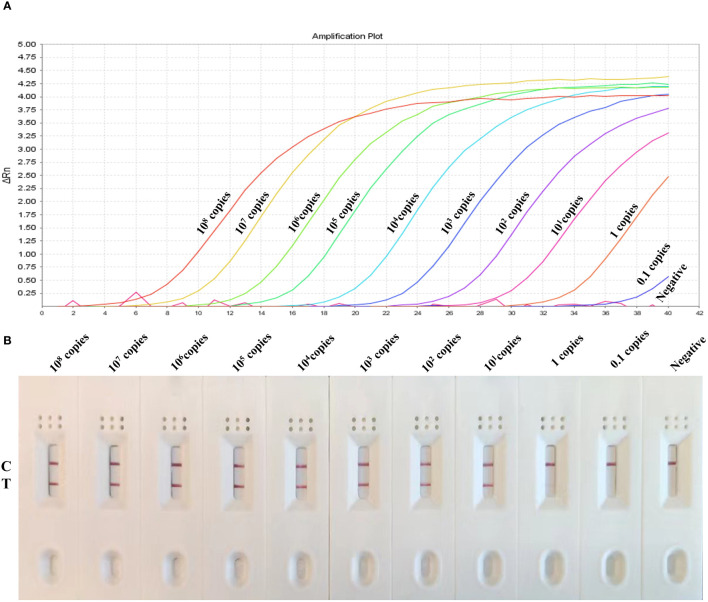
Sensitivity of the MIRA-LFD assay and the real-time PCR assay. **(A)** Serially diluted GAstV II plasmid of targeted gene (1×10^8^ copies/μL-0.1 copies/μL) was tested by real-time PCR. NC, negative control. **(B)** The quantity of gene amplification results was tested by MIRA-LFD. NC, RNase-free ddH_2_O, C, control line; T, test line.

For the sensitivity analysis, 10-fold serial dilutions of target gene ranging from 10^8^ to 0.1 copies/uL was used to evaluate the detection limit of MIRA-LFD and We compared the results with those obtained from the real-time PCR assay. The results revealed that the developed MIRA-LFD assay displayed a detection limit of 1 copies for GAstV II as indicated by a weak test line and the curves of 1 copies/μL plasmid with low fluorescence value in qRT-PCR, while the higher concentration produce intensity signals (**Figure 6**). However, the sample 0.1 copies/ul and the negative control (ddH_2_O) didn’t exhibit a test line. Additionally, no obvious fluorescence signal was detected in qRT-PCR assay (Ct > 36) for these samples. In summary, the sensitivity detection achieved by the MIRA-LFD assay was comparable to that of the real-time PCR assay. This suggests that the developed MIRA-LFD assay is suitable for the detection of GAstV II and can provide reliable results even at low target concentrations.

### 3.5 Evaluation with clinical samples by MIRA-LFD

To further evaluate the practicality of MIRA-LFD assay, 90 clinical samples were used for validation. The nucleic acid extraction of the samples were analyzed using both the GAstV II MIRA-LFD assay and the RT-qPCR assay. The detection results obtained from the two methods were compared. Among these clinical samples, all the 77 positive samples and 13 negative samples were accurately detected by both the MIRA-LFD assay and the RT-qPCR assay (**Table 2**). These results indicated that the established MIRA-LFD assay exhibits comparable detection accuracy to real-time PCR for clinical sample analysis. Moreover, the MIRA-LFD assay offers the advantage of being faster than RT-qPCR, highlighting its potential as a rapid and reliable diagnostic tool for GAstV II in clinical samples. The successful validation of the MIRA-LFD assay suggests its suitability for on-site testing, eliminating the need to send samples to a laboratory for analysis. This enhances the convenience and accessibility of GAstV II detection, particularly in settings where rapid diagnosis is crucial for disease management and control.

**Table 2:** Comparison of real-time PCR and MIRA-LFD results for clinical samples.

Results	MIRA-LFD positive	MIRA-LFD negative	Coincidence rate
qRT-PCR positive	77	0	100%
qRT-PCR negative	0	13	
Total	77	13	

## 4 Discussion

The emergence of GAstV as a significant pathogen causing clinical diseases in geese has resulted in substantial economic losses in the Chinese goose industry. Particularly, GAstV II has emerged as the predominant pathogen in recent years, causing severe infections in waterfowl, including various species of ducklings and geese. Epidemiological surveys have revealed frequent co-infections of GAstV II with goose parvovirus (GPV) in clinical samples (Liu et al., 2020). Currently, there are no effective commercial vaccines or treatments available to control GAstV II infections. Therefore, there is an urgent need to develop a sensitive, specific, and convenient detection method for the rapid and simple identification of GAstV II infection. Such a diagnostic tool would be invaluable for early detection, prompt management, and prevention of further spread of this emerging virus, ultimately helping to mitigate economic losses and protect the health of waterfowl populations.

With the rapid development of molecular biomedical technologies in recent years, several isothermal amplification solutions for both DNA and RNA have been advanced. These techniques involve a complex process of continuous transcription and reverse transcription between cDNA and RNA to achieve nucleic acid amplification. Among these techniques, MIRA stands out as an emerging constant temperature amplification technology with significantly improved efficiency compared to RPA. MIRA achieves exponential amplification in a short period at a constant temperature, offering several advantages over traditional PCR methods.

In MIRA, the first step involves the reverse transcription of RNA into dsDNA by reverse transcriptase (M-MLV RT). MIRA technology leverages the synergistic action of four functional proteins, including DNA helicase helicase, recombinase-RecA, single-stranded binding (SSB) protein, DNA polymerase (Piepenburg et al., 2006). This cooperative action facilitates rapid formation of the D-loop (Ji et al., 2022), allowing the reaction to be completed within 30 minutes at temperatures ranging from 25°C to 42°C, depending on experimental conditions. Unlike other isothermal amplification techniques such as RPA and RAA, which use T4 UvsX and E. coli UvsX recombinant enzymes, MIRA utilizes Streptomyces azure recA (SC-recA) recombinant enzyme, enhancing reaction stability and resistance to interference (Heng et al., 2022). enabling detection under various non-experimental conditions, particularly in field settings. Different studies on target gene detection using MIRA have shown slight variations in the optimal temperature, with 42°C generally being most suitable in most cases. In this assay for detecting GAstV II, we found that a temperature of 37°C also exhibited high reaction activity and similar sensitivity to 42°C. Therefore, both 37°C and 42°C can be utilized as reaction temperatures here. Considering that the RNA one-step MIRA relies on the high transcriptional activity of reverse transcriptase and based on manufacturer information that RT-nfo reagent achieves optimal activity for reverse transcription and amplification of trace RNA nucleic acids at 42°C, so 42°C was chosen for the subsequent assays. MIRA presents a promising approach for point-of-care testing (POCT), overcoming some limitations of PCR, which typically requires specialized equipment and at least an hour to complete.

**Figure 7:**
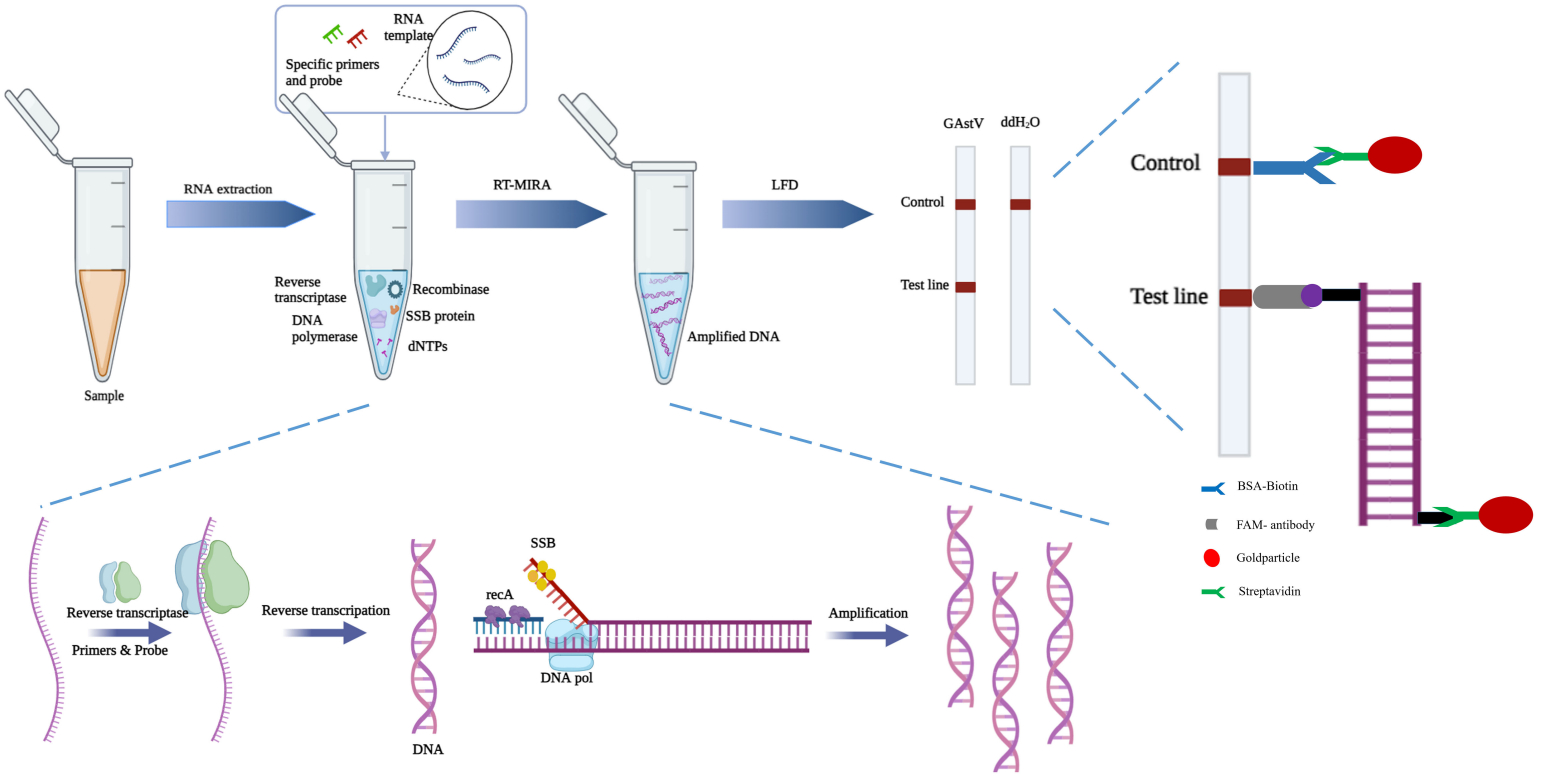
Scheme summarizing the entire process.

In addition, MIRA-LFD combines MIRA amplification with lateral flow dipstick (LFD) technology, offering several advantages such as short reaction times, ease of achieving reaction temperatures, and convenient visualization of results. LFD technology, known for its high sensitivity in detecting trace antigens, utilizes amplified products containing biotin to hybridize with probes labeled with 5-carboxyfluorescein (FAM) and bind to colloidal gold-labeled antibodies (Chen et al., 2020b; Sun et al., 2021). This results in intuitive detection results that can be observed directly with the naked eye. While PCR requires agarose gel electrophoresis to distinguish amplicons and gel imagers for visualization of results, real-time PCR allows for real-time detection of the entire amplification process through accumulation of fluorescence signals. Therefore, in this study, MIRA-LFD represents a novel multi-enzyme constant temperature rapid amplification combined with lateral chromatography strip technology. With the potential to be completed in just 20 minutes, MIRA-LFD offers a significant reduction in detection time and simplifies the testing process, making it a valuable tool for various diagnostic applications (**Figure 7**). Indeed, it’s important to note that while LFDs rely on capillary forces for their function, MIRA reaction products are thick liquids due to the various enzymes and reaction buffers used, which do not migrate well through LFD materials via capillary forces. Therefore, the MIRA reaction products were diluted with ddH_2_O. Additionally, since LFD materials from different companies have varying characteristics, products were diluted at a 1:20 ratio to ensure compatibility with a diverse range of LFD sources. thick liquids due to the various enzymes and reaction buffers used, which do not migrate well through LFD materials via capillary forces. The nucleic acid colloidal gold strip used for detection features colloidal gold labeled with streptavidin and a detection line labeled with anti-fluorescein antibodies. Streptavidin acts as a receptor, while biotin serves as a ligand. Anti-hydroxy-fluorescein antibodies bind to hydroxyl-fluorescein. When the MIRA products are applied to the LFD, biotin on the product binds to the colloidal gold modified with streptavidin. As the product reaches the detection line, anti-fluorescein antibody on the detection line captures the product labeled with hydroxyl fluorescein (FAM), resulting in a visible color change. Excess colloidal gold moves to the quality control line, where biotin binding also causes a color change. The size of the products primarily affects the intensity of the detection line color, not the migration speed. Prolonged observation beyond 5 minutes could lead to accumulation of primer dimers over time, potentially causing nonspecific binding that negatively impacts accuracy.

Since the initial identification of GAstV II in goslings, various PCR-based assays and isothermal amplification methods, including SYBR Green-based qPCR, TaqMan probe-based qPCR, and LAMP, have been developed (Wan et al., 2019; He et al., 2020; Ji et al., 2020). However, these methods often face challenges in meeting the requirements of grassroots detection and operational convenience, frequently requiring significant time for diagnosis. In our study, we introduced a novel one-step rapid assay for GAstV II detection by combining the innovative techniques of MIRA and LFD. Compared to RT-qPCR assays, which are considered the “gold standard” for identifying pathogenic microorganisms, MIRA-LFD assays offer several advantages. They do not require expensive instruments, and compared to other isothermal amplification-based assays such as LAMP, MIRA-LFD assays are easier to design. One challenge with LAMP assays is the potential formation of dimers between the six primers used in the reaction, which can lead to false-positive signals. Therefore, designing LAMP primers and avoiding dimer formation remains a significant challenge. Additionally, MIRA-LFD assays require less time than both RT-qPCR and LAMP assays, making them more suitable for rapid and on-site detection.

MIRA-LFD technology has been successfully applied to detect specific human pathogens, including Vibrio parahaemolyticus, SARS-CoV-2, and hepatitis C virus (Ghosh et al., 2022; Wang et al., 2022b; Park and Zhang, 2024). To our knowledge, this is the first application of MIRA-LFD assay to the detection of GAstV II. This study not only demonstrates the effectiveness of MIRA-LFD in detecting GAstV II but also lays the foundation for the development of MIRA-LFD for detecting other animal pathogens. This advancement has the potential to revolutionize pathogen detection in veterinary medicine, offering rapid, sensitive, and cost-effective diagnostic solutions.

The MIRA-LFD detection method established in this study offers several advantages for the rapid detection of GAstV II. It achieves amplification of a small amount of nucleic acid to a detectable level within 15 minutes at 42°C, with a minimum detection limit of 1 copy/μL, comparable to TaqMan PCR. This rapid turnaround time is crucial for controlling the spread of the virus, enabling timely intervention measures to be implemented. Furthermore, the MIRA-LFD detection method demonstrates high specificity, with a detection accuracy of 100% compared to TaqMan PCR. Importantly, it does not cross-react with other viruses commonly found in waterfowls, such as TMUV, GPV, CDRV, NDRV, NDV and so on. This high specificity ensures reliable identification of GAstV II, minimizing the risk of false-positive results and facilitating accurate diagnosis, providing a valuable tool for controlling the spread of the virus and safeguarding the health of waterfowl populations.

In summary, MIRA-LFD represents a significant advancement in pathogen detection, offering several advantages over conventional identification methods. Its rapid turnaround time, enhanced effectiveness, and heightened sensitivity make it particularly suitable for field applications, where timely detection is crucial for disease management. Furthermore, the simplicity and ease of use of the MIRA-LFD assay position it as a potential mainstream method for quantitative molecular detection of microbiological pathogens in the future. Its ability to deliver accurate results quickly and efficiently makes it an invaluable tool for researchers, clinicians, and field workers alike, facilitating prompt diagnosis and intervention to mitigate the spread of infectious diseases.

## Data availability statement

The datasets presented in this study can be found in online repositories. The names of the repository/repositories and accession number(s) can be found in the article/**Supplementary Material**.

## Ethics statement

The animal study was approved by the Institutional Animal Care and Use Committee (IACUC) of Zhejiang Academy of Agricultural Sciences (protocol code 2023ZAASLA10). The study was conducted in accordance with the local legislation and institutional requirements.

## Author contributions

YZ: Data curation, Funding acquisition, Investigation, Methodology, Resources, Software, Writing – original draft, Writing – review & editing. LC: Formal analysis, Investigation, Software, Writing – original draft. XX: Formal analysis, Investigation, Software, Writing – review & editing. WY: Investigation, Methodology, Writing – review & editing, Formal analysis. ZN: Data curation, Formal analysis, Software, Writing – review & editing. SH: Formal analysis, Software, Writing – review & editing. JH: Formal analysis, Investigation, Writing – review & editing, Methodology. TY: Formal analysis, Software, Writing – review & editing. HY: Formal analysis, Writing – review & editing. HW: Formal analysis, Investigation, Methodology, Writing – review & editing. CZ: Data curation, Formal analysis, Funding acquisition, Writing – review & editing.
