# Supplementary material for: Development of a multienzyme isothermal and lateral flow dipstick combination assay for the rapid detection of goose astrovirus II
Source: Front Cell Infect Microbiol. 2024 Aug 6;14:1424212. doi: 10.3389/fcimb.2024.1424212 (PMC11333440; doi:10.3389/fcimb.2024.1424212)
Supplement: Supplementary file 1 [file Table_1.docx]

**Table S1 AAstV strains used for sequence alignment**

| **Strain Name** | **Accession No.** | **Species** | **Strain Name** | **Accession No.** | **Species** |
| --- | --- | --- | --- | --- | --- |
| AHAU5 | MN428645 | GAstV | SCCD | MW340534 | GAstV |
| GD | MN428642 | GAstV | ZJC14 | OK571391 | GAstV |
| CXZ | MH807626 | GAstV | AVE52 | MH028405 | ANV |
| GTF-04 | MN068023 | GAstV | BJCP510-2 | MN732558 | ANV |
| HN1G | KY807085 | GAstV | G4260 | AB033998 | ANV |
| JSHA | MK125058 | GAstV | SH10 | HQ899774 | ANV |
| ZJLD | OK571389 | GAstV | HBLP717-1 | MN725025 | CAstV |
| JRMS | OK571390 | GAstV | CkP5 | KX397576 | CAstV |
| JSCZ | OL963555 | GAstV | GA2011 | JF414802 | CAstV |
| JSW1 | OL963557 | GAstV | C-NGB | NC012437 | DAstV |
| JSSQ | OL963556 | GAstV | CPH | KJ020899 | DAstV |
| JSW10 | OL963558 | GAstV | HB2015 | KY646154 | DAstV |
| XSSH | OL982613 | GAstV | SL1 | KF753804 | DAstV |
| JSZ29 | OL982615 | GAstV | SL4 | KF753806 | DAstV |
| JSCZ15 | OL982614 | GAstV | WF1201 | JX439643 | DAstV |
| SDPY | MH052598 | GAstV | YP2 | JX624774 | DAstV |
| SDTA | MN809622 | GAstV | AK98 | EU143843 | TAstV |
| SDXT | MN399857 | GAstV | TA1 | NC_002470 | TAstV |
| AHDY | MH410610 | GAstV | TX_00 | EU143850 | TAstV |
| FLX | KY271027 | GAstV | TAstV2_1999 | NC-005790 | TAstV |

**Table S2. Information of samples**

| **Goose species** | **Farms** | **Provinces** | **Days of age** | **Sample** | **No. of samples** | **GAstV-II positive rate** |
| --- | --- | --- | --- | --- | --- | --- |
| Landaise geese | 1 | Zhejiang | 70 | kidney | 3 | 3（100%） |
| Landaise geese | 1 | Zhejiang | 14 | kidney | 18 | 16（88%） |
| Zhedong White geese | 1 | Zhejiang | 0 | embryo | 10 | 8（80%） |
| Zhedong White geese | 1 | Zhejiang | 12 | kidney | 12 | 11（91%） |
| Taihu geese | 1 | Jiangsu | 8 | Liver, kidney | 7 | 3 (43%) |
| Taihu geese | 1 | Jiangsu | 6 | kidney | 10 | 9 (90%) |
| Jiangnan White geese | 1 | Jiangsu | 10 | Liver, kidney | 6 | 6（100%） |
| Jiangnan White geese | 1 | Jiangsu | 8 | Liver, kidney | 12 | 9 （75%） |
| Landaise geese | 1 | Jiangsu | 14 | kidney | 5 | 5（100%） |
| Taihu geese | 1 | Anhui | 13 | kidney | 7 | 7 (100%) |
| **Total** | 10 |  |  |  | 90 | 77（86%） |
